# Influenza A: Associated Severe Cholestatic Liver Injury in a Patient With Metabolic Dysfunction-Associated Steatotic Liver Disease (MASLD)

**DOI:** 10.7759/cureus.106524

**Published:** 2026-04-06

**Authors:** Aryan Neupane, Ashmita Chhetri, Mario Gonzalez, Ashima Ghimire, Ahmed Gohar

**Affiliations:** 1 Internal Medicine, North Alabama Medical Center, Florence, USA; 2 Critical Care, North Alabama Medical Center, Florence, USA

**Keywords:** cholestatic liver injury, influenza a, intrahepatic cholestasis, liver biopsy, sepsis-induced cholestasis

## Abstract

Influenza A infection typically causes mild hepatocellular liver enzyme elevations, while clinically significant cholestatic liver injury is rare. We report a case of severe intrahepatic cholestasis associated with Influenza A infection in a patient with pre-existing metabolic dysfunction-associated steatotic liver disease (MASLD). A 65-year-old female with MASLD and chronic prosthetic hip infection presented with confusion, hypoglycemia, and sepsis. One month prior, liver function tests were normal. On admission, laboratory evaluation demonstrated a cholestatic pattern with total bilirubin 3.6 mg/dL (direct 3.2 mg/dL), alkaline phosphatase (ALP) 300 U/L, and international normalized ratio (INR) 2.0. Rapid testing confirmed Influenza A infection. Imaging, including abdominal ultrasound and hepatobiliary iminodiacetic acid (HIDA) scan, showed a dilated common bile duct without obstruction but markedly impaired hepatic uptake and excretion. Despite biliary stenting, total bilirubin and ALP peaked at 8.5 mg/dL and 817 U/L. Liver biopsy revealed steatosis, lobular inflammation, cholestasis, and stage 2 fibrosis consistent with MASLD and superimposed acute cholestatic injury. With oseltamivir and supportive care, liver function tests gradually normalized. Influenza A may rarely precipitate severe intrahepatic cholestasis, particularly in patients with underlying steatotic liver disease.

## Introduction

Influenza A is primarily a respiratory pathogen but can also affect the heart, nervous system, and liver. In hospitalized patients, mild, transient transaminase elevations are common, whereas pronounced cholestatic injury is unusual [1]. When it occurs, distinguishing it from hepatocellular injury is clinically important, as cholestatic liver injury often prompts evaluation for biliary obstruction, autoimmune disease, or drug-induced liver injury (DILI), potentially leading to unnecessary investigations. Antiviral agents used to treat influenza, including oseltamivir, have been linked to rare DILI with cholestatic or mixed patterns [2,3]. This case aims to highlight an uncommon presentation and adds to the limited literature on influenza-associated cholestasis.

## Case presentation

A 65-year-old woman with a history of metabolic dysfunction-associated steatotic liver disease (MASLD), chronic tobacco use, and a persistent prosthetic hip infection presented with acute confusion and slurred speech. One month prior, she had been hospitalized for a multidrug-resistant urinary tract infection and chronic prosthetic hip infection, during which her liver enzymes and bilirubin were entirely normal. She had recently completed a six-week course of intravenous ertapenem and was at her neurological baseline until two days before admission.

Upon presentation, the patient was found to be severely hypoglycemic with a glucose level of 40 mg/dL (reference range 65-99 mg/dL) and was encephalopathic. Laboratory evaluation revealed a cholestatic pattern with total bilirubin 3.6 mg/dL (0-1.0 mg/dL), including direct bilirubin 3.2 mg/dL (0-0.9 mg/dL), alkaline phosphatase 300 U/L (45-122 U/L), AST 141 U/L (0-59 U/L), and ALT 70 U/L (0-50 U/L). Coagulation studies demonstrated an INR of 2.0 (0.8-1.2), and the patient was thrombocytopenic with a platelet count of 51 ×10³/mm³ (150-375 ×10³/mm³). Additional laboratory studies showed lactate 4.9 mmol/L (0.7-1.9 mmol/L) and albumin 2.1 g/dL (3.4-5.0 g/dL), while ammonia was within normal limits at 10 µmol/L (9-30 µmol/L). Key lab findings are presented in Table 1. Rapid viral testing was positive for influenza A and negative for SARS-CoV-2. Viral hepatitis serologies for hepatitis A, B, and C were negative. The patient was started on oseltamivir, dextrose-containing fluids, and empiric broad-spectrum antibiotics for suspected sepsis.

**Table 1:** Key laboratory findings at presentation.

Laboratory Test	Result	Reference Range
Glucose	40 mg/dL	65–99 mg/dL
Total Billirubin	3.6 mg/dL	0–1.0 mg/dL
Direct Billirubin	3.2 mg/dL	0–0.9 mg/dL
Alkaline Phosphatase (ALP)	300 U/L	45–122 U/L
Aspartate Aminotransferase (AST)	141 U/L	0–59 U/L
Alanine Aminotransferase (ALT)	70 U/L	0–50 U/L
Prothrombin Time (PT)	20.3 seconds	9.0-11.6 seconds
INR	2.0	0.8-1.2
Activated Partial Thromboplastin Time (APTT)	67 seconds	20-35 seconds
Platelets	51 ×10³/mm³	150–375 ×10³/mm³
Lactate	4.9 mmol/L	0.7–1.9 mmol/L
Albumin	2.1 g/dL	3.4–5.0 g/dL
Ammonia	10 µmol/L	9–30 µmol/L

**Figure 1:**
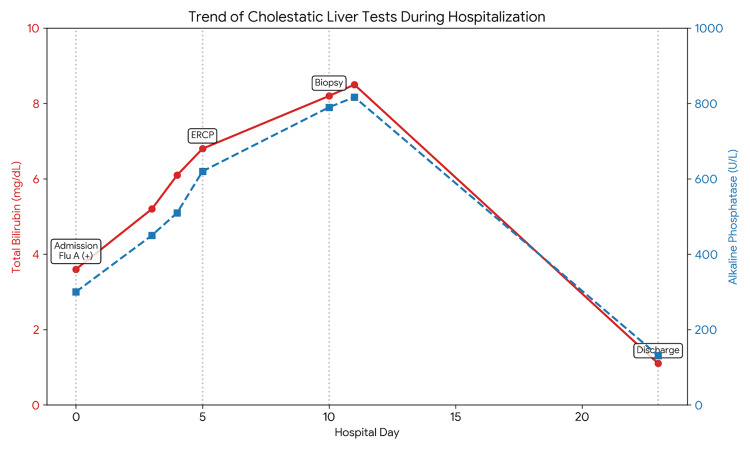
Trends in total bilirubin and alkaline phosphatase during hospitalization. Total bilirubin (solid line) and alkaline phosphatase (dashed line) demonstrate a parallel rise, peaking around hospital day 11, followed by a decline with clinical improvement. Key interventions, including ERCP (day 5) and liver biopsy (day 10), are indicated.

By hospital day three, her condition deteriorated with worsening mentation, hypoxia, and refractory hypotension requiring vasopressors and mechanical ventilation. A right upper quadrant ultrasound showed a fatty liver and a dilated common bile duct (CBD) of 16 mm (which was also a finding on previous imaging 2 years prior), though no obstructing stones were identified. A HIDA scan on day four further demonstrated poor hepatic uptake and absent biliary excretion, suggesting severe cholestasis with hepatocellular dysfunction [4]. The initial R factor was calculated at 0.69, quantitatively confirming a cholestatic pattern of liver injury (R < 2) and guiding the subsequent focus on biliary imaging and investigation for intrahepatic cholestasis [5]. To definitively rule out an obstruction, an endoscopic retrograde cholangiopancreatography (ERCP) was performed on day five; it confirmed a dilated CBD without stones or strictures. Despite a biliary sphincterotomy and stent placement, her bilirubin and ALP continued to rise, peaking at 8.5 mg/dL (predominantly direct) and 817 U/L, respectively (Figure 1).

On hospital day 10, a percutaneous liver biopsy was performed to investigate the worsening cholestasis. Histology revealed marked macrovesicular steatosis, lobular inflammation, and hepatocellular ballooning (NAFLD Activity Score of 7) with stage 2 fibrosis. Notably, there was prominent lobular cholestasis but no evidence of bile duct loss or "onion-skin" fibrosis. There was no plasma cell-rich portal infiltrate or interface hepatitis to suggest autoimmune hepatitis. Overall, the findings were interpreted as steatohepatitis (MASLD) with superimposed acute cholestatic injury, possibly viral- or drug-related [6-8].

Following the completion of oseltamivir and stabilization of her critical illness, the patient's liver function began to improve. On hospital day 11, she was extubated and weaned from vasopressors. Her biochemical markers steadily declined over the following two weeks. At the time of discharge on day 23, her total bilirubin had normalized to 1.1 mg/dL (0-1.0 mg/dL), and her ALP had improved to 131 U/L (45-122 U/L).

## Discussion

This patient developed severe intrahepatic cholestatic liver injury during Influenza A infection on a background of MASLD and septic shock, without evidence of mechanical obstruction and with autoimmune cholestatic disease considered very unlikely by imaging and biopsy. Most influenza-associated liver injury is mild and hepatocellular; the pronounced cholestasis, high bilirubin, and biopsy-proven lobular cholestasis seen here are unusual [1].

Hepatic involvement in influenza appears to be largely immune-mediated, driven by influenza-specific T-cell responses and Kupffer-cell activation rather than direct high-level viral replication in hepatocytes. Cytokines released during infection and sepsis can down-regulate hepatocellular and canalicular transporters, leading to impaired bile formation and cholestasis, as described in inflammation- and sepsis-induced cholestasis [9,10]. In this case, cholestasis peaked during vasopressor-dependent shock and improved in parallel with the resolution of systemic inflammation, supporting an inflammation-driven process [1,9,10].

Underlying MASLD likely contributed to the severity of cholestasis. Biopsy confirmed steatohepatitis with stage 2 fibrosis, indicating established chronic liver disease [6]. Patients with MASLD have altered bile acid handling and microvascular function, which increases susceptibility to additional insults such as infection, drugs, and hypoperfusion [7]. Critical illness and sepsis can trigger intrahepatic cholestasis through cytokine-mediated injury to hepatocytes and cholangiocytes, suggesting a “second-hit” effect in this patient [9,10].

Drug-induced liver injury was also considered. Ertapenem, meropenem, vancomycin, and oseltamivir have each been associated with rare liver injury. However, liver tests were normal throughout the six-week ertapenem course and remained normal for nearly a week after its cessation. Cholestasis was already present at admission, close to but before the start of oseltamivir, and worsened despite completion of antiviral therapy. These features make it unlikely that any single medication was the sole driver of injury, although a contributory role, especially in a vulnerable MASLD liver, cannot be excluded [2].

Mechanical obstruction and autoimmune cholestatic disease were also evaluated. The markedly dilated common bile duct initially raised concern for obstruction, but ultrasound and ERCP revealed no stones, sludge, strictures, or mass. The HIDA scan pattern of poor hepatic uptake and absent excretion is characteristic of severe cholestasis but does not reliably distinguish intrahepatic from extrahepatic obstruction [4]. Continued biochemical worsening after sphincterotomy and stenting, followed by improvement without further biliary intervention, made a primary mechanical cause unlikely. Autoimmune cholestatic disease was considered, but the absence of characteristic imaging findings and lack of chronic cholangitic or ductopenic changes on biopsy strongly argued against primary sclerosing cholangitis or primary biliary cholangitis [8,10].

Overall, this case highlights that influenza A can rarely present with a predominantly cholestatic pattern of liver injury, particularly in patients with MASLD and severe systemic inflammation [1]. In critically ill patients exposed to multiple potentially hepatotoxic medications, careful temporal correlation of drug exposure, clinical course, imaging, and histology is essential [9]. The most accurate description of this case is influenza-associated intrahepatic cholestatic liver injury superimposed on MASLD and critical illness, with possible but unproven contributions from oseltamivir and broad-spectrum antibiotics.

## Conclusions

Severe cholestatic liver injury is an uncommon manifestation of influenza A infection. In this case, the temporal relationship between systemic illness and rising cholestatic markers, the absence of structural biliary obstruction, and the liver biopsy demonstrating lobular cholestasis on a background of metabolic dysfunction-associated steatotic liver disease (MASLD) support a predominantly intrahepatic process, likely driven by systemic inflammation. However, this presentation is best understood as multifactorial. Although drug-induced liver injury remains a consideration, careful review of medication exposure in relation to liver test trends did not strongly support a single offending agent. In addition, certain diagnostic studies, including hepatitis E serology, gamma-glutamyl transferase, and a complete autoimmune hepatitis panel, were not obtained and represent limitations in fully excluding alternative etiologies. Nevertheless, the overall clinical course and improvement with resolution of the underlying illness support influenza A-associated cholestasis as a contributing factor. This case highlights the diagnostic challenges in distinguishing viral-associated cholestasis from other causes and underscores the importance of integrating clinical course, medication timing, and histologic findings.
